# The usefulness of case reports in managing emerging infectious disease

**DOI:** 10.1186/1752-1947-5-194

**Published:** 2011-05-20

**Authors:** Viroj Wiwanitkit

**Affiliations:** 1 Wiwanitkit House, Bangkhae, Bangkok, 10160, Thailand

## Abstract

Emerging infectious diseases are an important problem in medicine. Case reports usually document episodes in the early emerging phase or in a small outbreak. Although the case report is considered weak evidence in medical literature, it is usually the first report when there is a new emerging infectious disease. There is no doubt that case reports can provide useful information for further case series, reviews and studies. This editorial focuses on the usefulness of the case report on emerging infectious disease to the medical society. Publication in this area is highly welcomed by the journal and can serve as a future point of reference.

## 

The case report is a specific kind of publication in the medical literature. This specific kind of article is seldom seen in other non-medical literature. The case report is usually a short article describing "an interesting case" in medicine [1]. This might be a new medical condition, a new diagnostic approach, a new treatment or a new disease. In general, case reports usually describe a specific group of medical disorders including metabolic diseases, malignancies, infections and genetic disorders.

Case reports on infection have a long history. Some case reports can be found in the ancient literature. A good example is the description of a condition that is believed to be "malaria" in the papyrus [2]. In ancient Chinese and Egyptian papers, a condition that is believed to be "syphilis" is also recorded [3]. Hence, there is no doubt that infection has been focused on by physicians and medical scientists for many centuries.

Concerning the nature of case reports on infection, a variety of reports can be seen. For example, reports can be on new disease, a rare disease, a new diagnostic tool or a new treatment. An interesting type of case report is that of an emerging infectious disease. By definition, an emerging infectious disease is a condition of outbreak or epidemic of an infection [4]. This can be a newly detected disease or the reemergence of an old disease. Emerging infectious disease is a condition that needs urgent management, and it is a public health focus.

In the early emerging phase or in a small outbreak, the case reports are usually the first documents on those problems. A good example is the first case report from Mexico on the influenza of 2009 or swine flu before the present pandemic [5]. This outbreak report brought global attention to the possible emergence of this new serious infection [5]. Later, the infection became a pandemic around the world. Hundreds of publications on swine flu were published following this first case report. Of interest, the first case report in each country is usually the starting point to draw attention from local physicians and medical scientists. For example, the first case report on swine flu by Grasselli *et al*. [6] promoted generalized awareness of swine flu in Italy. Another similar scenario is the report on the outbreak of bird flu, another problematic atypical infection [7]. This report was also published before the existence of the outbreak of bird flu in many Asian countries.

Case reports cannot have as high scientific merit as well-designed, randomized clinical trials or meta-analyses. However, if the case report is well reviewed and screened before publication, it can be reliable [8]. Nevertheless, although the case report is considered weak evidence in the medical literature, it is usually the first report when there is a new emerging infectious disease. A case report on an emerging infectious disease is a signal emerging from the background noise. It is not possible to perform good research on the new emerging infection at its first appearance, since no one completely knows the details of that infection. During the early period, the focus is usually on controlling the new disease. In addition, good research on the emerging infectious disease usually takes a long time to perform and a longer time for publication of the results to occur. Hence, the data in case reports are useful information for planning corresponding to the emerging infection. The recommendation for treatment is usually set on the basis of the clinical information that can be derived from published case reports. A good example is the setting of the new severe acute respiratory syndrome (SARS) preparedness program based on the analysis of case reports on SARS [9]. However, having a case report peer-reviewed and published in a journal in the classical way might still take a long time. The present rapid online publication policy of the *Journal of Medical Case Reports *(*JMCR*) can help reduce delays in publication of interesting emerging infectious diseases. For example, *JMCR *has published an interesting first case report on an infection associated with the Panola Mountain *Ehrlichia *species [10].

In medicine, analysis of case reports can provide useful information for the general practitioner on the way an outbreak has emerged. New lessons might be learned. In terms of research, the case report can contribute to better understanding of a new and emerging infectious disease through a case series review. Publication in this area is highly welcomed by the journal and can serve as a future point of reference.

## Competing interests

VW is on the editorial board of the *Journal of Medical Case Reports*.
